# Efficacy and Safety of Chemotherapy after Immunotherapy in Patients with Advanced Non-Small-Cell Lung Cancer

**DOI:** 10.3390/jcm13133642

**Published:** 2024-06-21

**Authors:** Andrea Camerini, Francesca Mazzoni, Vieri Scotti, Carmelo Tibaldi, Andrea Sbrana, Luana Calabrò, Enrico Caliman, Lucia Pia Ciccone, Laura Bernardini, Jessica Graziani, Maria Antonietta Grosso, Antonio Chella, Giacomo Allegrini, Domenico Amoroso, Editta Baldini

**Affiliations:** 1 Medical Oncology, Versilia Hospital, Azienda USL Toscana Nord-Ovest, 55041 Lido di Camaiore, Italy; maria.antonietta.gro@gmail.com (M.A.G.); domenico.amoroso@uslnordovest.toscana.it (D.A.); 2 SODc Oncologia Medica, Azienda Ospedaliero Universitaria Careggi, 50134 Firenze, Italy; francescamazzoni@hotmail.com (F.M.); enrico.caliman@gmail.com (E.C.); 3 Radiation Oncology Unit, Oncology Department, Azienda Ospedaliero Universitaria Careggi, 50134 Firenze, Italy; vieri.scotti@unifi.it (V.S.); luciapia.ciccone@gmail.com (L.P.C.); 4 Medical Oncology, San Luca Hospital, Azienda USL Toscana Nord-Ovest, 55100 Lucca, Italy; carmelo.tibaldi@uslnordovest.toscana.it (C.T.); editta.baldini@uslnordovest.toscana.it (E.B.); 5 Pneumo-Oncology Unit, Azienda Ospedaliero-Universitaria Pisana, 50134 Pisa, Italy; andreasbrana89@gmail.com (A.S.); anto.kell@tiscali.it (A.C.); 6 Medical Oncology, Azienda Ospedaliera Universitaria Senese, 53100 Siena, Italy; 7 UO Oncologia Medica 2 Universitaria, Ospedale S. Chiara, Azienda Ospedaliero-Universitaria Pisana, 50134 Pisa, Italy; laura.bernardini94@gmail.com (L.B.); jess.graziani@gmail.com (J.G.); 8 Medical Oncology, Spedali Riuniti Livorno, Azienda USL Toscana Nord-Ovest, 57124 Livorno, Italy; giacomo.allegrini@uslnordovest.toscana.it

**Keywords:** NSCLC, immunotherapy, chemotherapy, neutrophil/lymphocyte ratio, second-line, single agent

## Abstract

**Background:** There are currently few data about the safety and effectiveness of chemotherapy for patients with metastatic non-small-cell lung cancer (NSCLC) who have progressed from prior immunotherapy. **Methods:** Data from patients with consecutive stage IIIB–IV, ECOG performance status (PS) 0–2, non-small-cell lung cancer (NSCLC) treated with combination or single-agent chemotherapy following progression on an earlier immunotherapy regimen were retrospectively gathered. Recorded were baseline attributes, outcome metrics, and toxicities. The neutrophil/lymphocyte (N/L) ratio’s predictive usefulness was examined through an exploratory analysis. **Results:** The analysis comprised one hundred subjects. The adeno/squamous carcinoma ratio was 77%/23%, the M/F ratio was 66%/34%, the ECOG PS was 0/1/≥2 47%/51%/2%, and the median PD-L1 expression was 50% (range 0–100). The median age was 67 (range 39–81) years. Prior immunotherapy included a single-agent treatment in 83% of cases, with pembrolizumab use being prevalent, and a median N/L ratio of four prior to chemotherapy. The overall median time-to-progression on previous immunotherapy was 6 months. After immunotherapy, just 33% of subjects underwent chemotherapy. A median of 4 (range 1–16) cycles of chemotherapy were administered; platinum doublets (primarily carboplatin) were delivered in only 31% of cases, vinorelbine accounted for 25%, taxanes for 25%, and gemcitabine for 8%. The median clinical benefit was 55%, while the overall response rate was 21%. The median overall survival was 5 months (range 1–22) and the median time to progression was 4 months (range 1–17). Subgroups with low and high N/L ratios were compared, but there was no discernible difference in survival. **Conclusions:** After immunotherapy, a small percentage of patients with advanced NSCLC had chemotherapy. Following immunotherapy advancement, chemotherapy demonstrated a moderate level of therapeutic effectiveness; no adverse concerns were noted. The effectiveness of chemotherapy following immunotherapy was not predicted by the baseline N/L ratio.

## 1. Introduction

Lung cancer is the second most frequent malignancy in both men (12%) and women (13%), and it is the leading cause of cancer death worldwide in both genders, accounting for more deaths than breast cancer, prostate cancer, and colorectal cancer combined [1]. The 5-year survival of lung cancer patients is 18% for men and 25% for women, burdened by the large proportion of patients with diagnosis of advanced stage disease [2].

Outcomes of advanced disease, however, have dramatically improved in the last few years with the development of molecular target therapy and immune checkpoint inhibitors (ICIs), thanks to the investigation of the molecular mechanisms underlying tumor biology. 

Immune checkpoint inhibitors, single-agent or combined with chemotherapy (ChT) [3,4,5,6,7], has become the new standard of care in patients with non-oncogene-addicted advanced lung carcinoma. The addition of ICIs to chemotherapy resulted in a significantly higher rate of overall survival (OS) and progression-free survival (PFS), with suitable safety. In non-oncogene-addicted advanced or metastatic non-small-cell lung cancer (NSCLC), ICIs plus chemotherapy represents a standard treatment option across each of the PD-L1 strata [3,4,5,6,7,8], while ICI single-agent treatment represents the standard of care for NSCLC with PD-L1 expression ≥ 50% [8,9,10,11]. 

However, evidence about the safety and efficacy of ChT as second- or third-line therapy after immunotherapy is poor, even if it is often proposed to patients. Additionally, many trials are currently evaluating potential new target therapies [12], but clinical studies investigating optimal treatment after disease progression on first- or second-line immunotherapy are still lacking. Also, pharmacological agents commonly used in palliative settings include several drugs, but consensus on the best systemic therapy has not been reached.

Actually, there is a rising need to identify predictive and prognostic factors of responses to ChT, in order to select patients who are likely to benefit from ChT administered as a second or subsequent line.

Therefore, we aimed to evaluate, with a multicenter retrospective observational study, our experience in treatment sequencing after first-line immunotherapy, and assessed different drugs used, side effects, and expected benefits to patients.

## 2. Patients and Methods 

### 2.1. Patient Selection

We retrospectively collected a cohort of consecutive patients with diagnoses of NSCLC stage IIIB–IV receiving an active treatment in five different oncology centers in Tuscany (Italy) in a two-year period between 2021 and 2023. All patients were treated with single-agent or platinum combination ChT after progression on immunotherapy, which must have been the immediately previous regimen administered. After verification of a patient’s eligibility, the investigator informed the subject (in the presence of a legal representative, if requested) in detail about this study, its methodological aspects, and purpose. Special care was taken to underline that participation in this study was voluntary and without an impact on the treatment of the disease. Informed consent was obtained from all subjects involved in this study. The protocol received approval by the institutional ethical committee “Comitato Etico di Area Vasta Nord Ovest (CEAVNO)” on 27 July 2023, identification n°24740. 

Main inclusion criteria were as follows: age ≥ 18; ability to provide written informed consent; diagnosis of NSCLC cytologically or histologically confirmed; radiologic diagnosis of stage IIIB–IV disease; availability of tumor assessment during follow up; and access to clinical information, pathological features, and survival data. Patients were excluded from this study if they received prior therapy with an antiPD-1, antiPD-L1, or an agent directed to another stimulatory or coinhibitory T-cell receptor before the immunotherapy under study; received any prior systemic anticancer therapy for a known additional malignancy within the past 5 years; had a history or current evidence of any condition that might confound the results of this study or interfere with their participation for the full duration of this study; or had a known psychiatric or substance abuse disorder that would interfere with their ability to cooperate with the requirements of this study. 

Baseline characteristics, outcome measures, and toxicities were recorded. Patients were treated and monitored in clinical practice. Written informed consent from patients for research use of data was obtained before the investigation. All clinical data were collected from medical records at each center and, when not available, from phone calls or central registry consultations (the last for survival status updates only). The response to treatment was assessed as per standard clinical practice and according to RECIST 1.1 by local investigators; complete response (CR), partial response (PR), stable disease (SD), and progressive disease (PD) were defined according to RECIST criteria. Safety was assessed at each planned clinical visit and registered according to Common Terminology Criteria for Adverse Events version 5.0 (CTCAE v. 5.0).

Analyses were performed according to clinical practice. Data were collected and analyzed assuring patient anonymization at every stage of data processing. PD-L1 tumor proportion scores were evaluated by immunohistochemistry in all samples at the local pathology laboratory level according to internally validated protocols. No additional tests (blood, pathology, etc.), other that those of usual clinical practice, were required at any time during this study. An exploratory analysis of the predictive value of the neutrophil/lymphocyte (N/L) ratio was performed.

### 2.2. Statistical Analyses

The primary aim of this study was to assess the efficacy and safety of ChT delivered as immediate-next treatment after progression on ICIs-based immunotherapy in patients with pre-treated advanced NSCLC. The secondary exploratory aim of this study was to investigate the prognostic and predictive role of the N/L ratio in terms of outcomes defined as time-to-progression (TTP) and OS. For this analysis, the following parameters were recorded: age at diagnosis; Eastern Cooperative Oncology Group performance status (ECOG PS 0-2) at diagnosis; sex; histology; stage according to TNM VIII edition (IIIB–-IV); smoking habits; histologic features; median PD-L1 tumor proportion score % expression (range); laboratory values (white blood cell (WBC) count, absolute neutrophil count (ANC), lymphocytes, monocytes, platelets, and lactate dehydrogenase (LDH)); duration and type of chemotherapy; immunotherapy as single-agent or combination administered before chemotherapy; treatment lines; number of cycles of therapy; hematological and non-hematological chemotherapy-related toxicities graded according to CTCAE v. 5.0; response evaluation criteria (RECIST 1.1) outcome; molecular profile (EGFR, ALK, KRAS, ROS1, and BRAF status (mutated/wild, type/unknown)) according to the local laboratory; TTP; OS; second-line TTP; second-line OS; third-line TTP; and third-line OS. Categorical data are presented as percentages for the entire population, continuous data are reported as the mean and range or median and range for normally or skewed distributed data, respectively. The Kaplan–Meier method was used to estimate OS, PFS, and TTP. Data for patients alive or lost to follow-up were censored for OS at the time of last follow-up. The stratified log-rank test was used to assess differences between subgroups with low vs. high N/L ratios of TTP. Missing data were not included in the models. All tests were two-sided, with a *p*-value of < 0.05 considered significant. Data were analyzed with SPSS version 25.0 (IBM Corp., Armonk, NY, USA).

## 3. Results

The analysis included 100 patients, with baseline characteristics reported in Table 1. The median age at diagnosis was 67 years (range 39–81); 66% of patients were male and 34% were female; 98% of the population had an ECOG PS of 0–1; the majority of patients had stage IV NSCLC (85%); most patients were former or current smokers; adenocarcinoma was more frequent than squamous cell carcinoma (77% vs. 23%, respectively); the median PD-L1 tumor proportion score was 50% (range 0–100%); EGFR mutated status recurred in 3% of patients; ALK rearrangements occurred in 1% of patients; KRAS mutations occurred in 26% of patients; ROS1 rearrangement occurred in no patients; and BRAF mutation occurred in only one patient. Clinical characteristics of immunotherapy treatment delivered immediately before chemotherapy are reported in Table 2. Most patients received pembrolizumab administered intravenously at a dose of 200 mg every 3 weeks before chemotherapy (58% of patients) and were treated with single-agent ICI (83% vs. 17%); first-line setting of immunotherapy was prevalent (56% vs. 31% for second-line and 13% for third-line). According to RECIST criteria, 29% of patients achieved PR, 42% achieved SD, and 29% achieved PD; no CR were reported.

Characteristics of chemotherapy treatments administered are reported in Table 3.

The most commonly administered drugs were a carboplatin doublet (22% of patients) and vinorelbine (25%), followed by taxanes (17%) and a cisplatin doublet (9%). Carboplatin-based doublets were administered intravenously (carboplatin area under the concentration curve AUC 4–6 on day 1 or AUC 2 on days 1, 8, and 15) every 21 days; cisplatin-based doublets were administered intravenously (cisplatin 75 mg/mq on day 1) every 21 days; docetaxel 75 mg/mq was administered intravenously on day 1 every 21 days, or 33 mg/mq on days 1 and 8 every 21 days; and nintedanib 200 mg was administered orally, twice daily, on days 2–21 of each 21-day cycle. Other drugs delivered were taxanes (paclitaxel 175–200 mg/mq on day 1 every 21 days, or 60–80 mg/mq on days 1, 8, and 15 every 21 days) administered intravenously; vinorelbine 60 mg/mq on days 1 and 8 every 21 days, or with a metronomic schedule administered orally, or vinorelbine 25–30 mg/mq on days 1 and 8 every 21 days administered intravenously; gemcitabine 1000–1250 mg/mq on days 1 and 8 every 21 days administered intravenously; and pemetrexed 500 mg/mq on day 1 every 21 days administered intravenously. The most frequent setting was second-line, and PR RECIST was observed in 20% of patients in front of 45% of PD RECIST. The median TTP was 4 months (range 1–17), and the median OS was 5 months (range 1–22).

Precisely in second-line (2L) settings, the median TTP 2L observed was 4 months (range 1–12) and the median OS 2L was 5 months (range 1–22). The median TTP in third-line settings and beyond were 4 months (range 1–17) and 5 months (range 1–19), respectively, and 76% of patients received only the best supportive care (BSC) beyond.

All grade chemotherapy-related hematological and non-hematological toxicities are presented in Table 4. The most common all grade adverse event was hemoglobin decrease (42%), followed by ANC decrease (26%) and nausea and vomiting (25%). The most serious adverse event (grade 3–4) was ANC decrease. 

Table 5 shows median baseline blood cells counts and LDH values. The median ANC value was 5.22 (range 2.55–15.13) × 10^3^/mcl, the median lymphocytes value was 1.37 (range 0.40–5.00) × 10^3^/mcl, the median monocytes value was 0.59 (range 0.90–4.10) × 10^3^/mcl, and the median LDH value was 240 (range 119–902) U/L. The N/L ratio was not related to outcomes, and no significant difference in survival was reported between subgroups with low vs. high N/L ratios.

## 4. Discussion

Second-line ChT after progression to first-line immunotherapy is currently an available option for NSCLC patients regardless of PD-L1 expression, but it could be burdened by a high rate of adverse events with a modest improvement in survival as compared with best supportive care (BSC) [13]. Although the benefit of systemic first-line immunotherapy has been widely demonstrated, the second-line treatment of choice has yet to be defined, and the role of palliative chemotherapy is not yet clear. Data about second-line therapies derived from historical trials that did not include actual standard of care in first-line settings [14] were influenced by the advent of the practice of changing ICIs. Regardless, the new treatment algorithm for advanced NSCLC have to consider previously novel immunotherapeutic agents and target therapies, not only in first- or second-line settings of treatment, but recently, also in neoadjuvant [15] and adjuvant settings [16,17], and maybe there is a small margin for ChT.

According to the latest ESMO guidelines [8], next-line treatment strategy is impacted by the treatment given in the previous line, and ChT should be considered only for patients with a PS 0–2 without major comorbidities.

Treatments currently administered after immunotherapy are derived from analyses that considered only platinum-based chemotherapy as the previous standard of care, i.e., docetaxel [18,19] and its association with nintedanib [20], which achieved an OS of 12.6 months and a PFS of 4.4 months, or pemetrexed [21]. In addition, even if prospective trials for third-generation drugs (such as gemcitabine, taxanes, and vinorelbine) showed improved survival outcome for NSCLC patients [22], their population does not match with the real-world cohort, and < 30% of our clinical cases meet the eligibility criteria of these trials [23,24]. 

These analyses are lacking about which therapeutic approach is better after disease progression while on immunotherapy: some studies showed a possibly enhanced activity of ChT when used at progression from ICIs [25,26], but evidence is weak, and randomized trials are missing.

There are limitations associated with the retrospective design, including missing data, unaccounted bias in patient selection, sampling bias, and subjective assessments (e.g., ECOG PS or evaluation of clinical adverse events). When multiple adverse events occurred in the same patient, only the most clinically significant event was recorded. Therefore, reported toxicities may have been underestimated. Another limitation of our study is the relatively small sample size, which may have affected the statistical power and generalizability of our findings. However, it should be noted that this study reflected a real-life setting obtained from a homogeneous population. The limited follow-up period should not have significant implications due to the absence of long-term outcomes in this group of pre-treated patients.

Our results match with other recently conducted analyses. As observed by Liu et al. [27] and Auclin et al. [28], survival outcomes obtained with ChT after immunotherapy were consistent with historical OS data before the availability of ICIs; our data show that a synergistic effect between immunotherapy and ChT is unlikely. The OS we found in our cohort is lower than that shown in most second-line studies [20], but it was affected by the inclusion of patients with factors known to be indicative of poor prognosis (e.g., inclusion of ECOG 2, older mean age, and exclusion of stages below IIIB).

In our small cohort, however, ChT after progression to ICIs seemed to be suitable, and no differences in safety were found between our data and historical data; therefore, immunotherapy may not enhance the toxicity of ChT. It is important to identify predictive and prognostic factors of responses to ChT after immunotherapy, in order to assess if the benefit of ChT is influenced by the type of molecular driver, if immunotherapy in oncogene-addicted NSCLC affects the response to therapy, or if the response itself may be influenced by the level of PD-L1 expression.

Moreover, in order to enhance traditional clinical trial results with our real-world experience, we underline that in our study, safety in ChT after immunotherapy was not more burdened than it was in historical studies, and that the N/L ratio did not affect outcome; assessing the risk/benefit ratio on a case-by-case basis still remains the most important choice.

Pre-treated patients with NSCLC were often precluded from further treatment because of clinical deterioration [13], and only a minority of advanced NSCLC patients received chemotherapy after immunotherapy. Chemotherapy showed modest clinical efficacy after progression on immunotherapy, while no safety issues were recorded. The baseline N/L ratio failed to predict chemotherapy benefits after immunotherapy. 

Based on our results, ChT should be considered in patients with a PS 0–2 without major comorbidities, treatment has to be carefully discussed and customized regarding disease characteristics, and patients’ clinical features and the selection of responder patients are still a challenge to be faced.

## 5. Conclusions

The rapid immunotherapy shift to a first-line setting causes a therapeutic “black-hole” in second- and later-line settings that is even more relevant in the actual scenario of ICIs use in early stage disease. In the absence of any treatment proven to be effective in such a challenging scenario, clinicians are forced to use classic chemotherapy regimens, but with limited efficacy. Our data confirm the modest activity of standard chemotherapy and highlight (one more time) the need for specific clinical trials to fill this gap.

## Figures and Tables

**Table 1:** Study population’s characteristics (*n* = 100).

Median age [range]	67 (39–81) yrs
Male/Female (*n*/%)	66/34 (66%/34%)
ECOG PS 0/1/2 (*n*/%)	47/51/2 (47%/51%/2%)
Stage (IIIB/IV)	15/85 (15%/85%)
Smoking habit * (N/P/C)	9/47/40 (9%/47%/40%)
Histology (ADK/SQK)	77/23 (77%/23%)
Median PD-L1% * [range]	50% (0–100)
EGFR status (mut/wt/missing)	3/84/13 (3%/84%/13%)
ALK status (mut/wt/missing)	1/82/17 (1%/82%/17%)
KRAS status (mut/wt/missing)	26/39/35 (26%/39%/35%)
ROS1 status (mut/wt/missing)	0/67/33 (0%/67%/33%)
BRAF status (mut/wt/missing)	1/46/53 (1%/46%/53%)

N = never; P = past; C = current; ADK = adenocarcinoma; SQK = squamous cell carcinoma; mut = mutated; wt = wild type. * Some values are missing.

**Table 2:** Clinical characteristics of immunotherapy treatment delivered immediately before chemotherapy (*n* = 100).

Drug Delivered (*n*/%)	
Pembolizumab 200 mg every 3 weeks	58 (58%)
Nivolumab 240 mg every 2 weeks	33 (33%)
Atezolizumab 1200 mg every 3 weeks	5 (5%)
Other *	4 (4%)
Single/Combo **	83/17
Treatment line 1/2/3+ (*n*/%)	56 (56%)/31 (31%)/13 (13%)
Median n° cycles [range]	10 (2–44)
Outcome	(*n*/%)
CR	0 (0%)
PR	29 (29%)
SD	42 (42%)
PD	29 (29%)
Median TTP [range]	6 (2–24) months

CR = complete response; PR = partial response; SD = stable disease; PD = progressive disease; TTP = time to progression. * Other = durvalumab (2) and avelumab (2); ** all combinations were pembrolizumab-based.

**Table 3:** Clinical characteristics of chemotherapy treatments delivered (*n* = 100).

Drug Delivered * (*n*/%)	
Cisplatin doublet	9 (9%)
Carboplatin doublet	22 (22%)
Docetaxel + Nintedanib	8 (8%)
Taxanes	17 (17%)
Vinorelbine	23 (25%)
Gemcitabine	8 (8%)
Pemetrexed	1(1%)
Treatment line 2/3/4+	64/31/5
Median n° cycles [range]	4 (1–16)
Outcome (*n*/%)	
CR	1 (1%)
PR	20 (20%)
SD	34 (34%)
PD	45 (45%)
Median TTP [range]	4 (1–17) months
Median OS [range]	5 (1–22) months
Median TTP 2L [range]	4 (1–12) months
Median OS 2L [range]	5 (1–22) months
Median TTP 3L+ [range]	4 (1–17) months
Median OS 3L+ [range]	5 (1–19) months
Further line (Yes/No)	24/76

CR = complete response; PR = partial response; SD = stable disease; PD = progressive disease; TTP = time to progression; OS = overall survival; 2L = second-line; 3L+ = third or further line. * Some values are missing.

**Table 4:** All grade, mild (grade 1–2), and serious (grade 3–4) chemotherapy-related hematological (upper panel) and non-hematological (lower panel) toxicities (*n* = 100).

	All Grade	Grade 1–2	Grade 3–4
White blood cells count decrease	23%	18%	5%
Absolute neutrophil count decrease	26%	15%	11%
Hemoglobin decrease	42%	33%	9%
Platelet count decrease	13%	8%	5%
Fatigue	11%	9%	2%
Diarrhea	8%	8%	0%
Oral mucositis	16%	15%	1%
Nausea and vomiting	25%	21%	4%

**Table 5:** Median baseline (before chemotherapy) blood cells counts and lactate dehydrogenase values (upper panel); relationship between neutrophil/lymphocyte ratio and treatment outcomes (lower panel) (*n* = 100).

Median WBC * [range]	7.80 (4.10–19.20) × 10^3^/mcl
Median ANC * [range]	5.22 (2.55–15.13) × 10^3^/mcl
Median lymphocytes * [range]	1.37 (0.40–5.00) × 10^3^/mcl
Median monocytes * [range]	0.59 (0.90–4.10) × 10^3^/mcl
Median Platelets * [range]	287 (110–540) × 10^3^/mcl
Median LDH * [range]	240 (119–902) U/L
Median N/L ratio	4
Mean TTP (low vs. high) 3.7 ± 2.0 vs. 3.3 ± 2.5	*p* = ns
Mean OS (low vs. high) 5.7 ± 3.1 vs. 4.7 ± 2.9	*p* = 0.1 ns

WBC = white blood cells; ANC = absolute neutrophil count; LDH = lactate dehydrogenase; N/L = neutrophil/lymphocyte ratio; TTP = time to progression; OS = overall survival; ns = not significant. * Some values are missing.

## Data Availability

Data presented in this study are available on request from the corresponding author. Data are not publicly available due to institutional policy.

## References

[1] Siegel R.L., Miller K.D., Wagle N.S., Jemal A. (2023). Cancer statistics, 2023. CA Cancer J. Clin..

[2] American Cancer Society Cancer Facts & Figures 2022. https://seer.cancer.gov/statistics-network/explorer/application.html?site=1&data_type=1&graph_type=2&compareBy=sex&chk_sex_3=3&chk_sex_2=2&rate_type=2&race=1&age_range=1&hdn_stage=101&advopt_precision=1&advopt_show_ci=on&hdn_view=0&advopt_show_apc=on&advopt_display=2#resultsRegion0.

[3] Gandhi L., Rodríguez-Abreu D., Gadgeel S., Esteban E., Felip E., De Angelis F., Domine M., Clingan P., Hochmair M.J., Powell S.F. (2018). Pembrolizumab plus Chemotherapy in Metastatic Non-Small-Cell Lung Cancer. N. Engl. J. Med..

[4] West H., McCleod M., Hussein M., Morabito A., Rittmeyer A., Conter H.J., Kopp H.-G., Daniel D., McCune S., Mekhail T. (2019). Atezolizumab in combination with carboplatin plus nab-paclitaxel chemotherapy compared with chemotherapy alone as first-line treatment for metastatic non-squamous non-small-cell lung cancer (IMpower130): A multicentre, randomised, open-label, phase 3 trial. Lancet Oncol..

[5] Paz-Ares L., Ciuleanu T.-E., Cobo M., Schenker M., Zurawski B., Menezes J., Richardet E., Bennouna J., Felip E., Juan-Vidal O. (2021). First-line nivolumab plus ipilimumab combined with two cycles of chemotherapy in patients with non-small-cell lung cancer (CheckMate 9LA): An international, randomised, open-label, phase 3 trial. Lancet Oncol..

[6] Paz-Ares L., Luft A., Vicente D., Tafreshi A., Gümüş M., Mazières J., Hermes B., Çay Şenler F., Csőszi T., Fülöp A. (2018). Pembrolizumab plus Chemotherapy for Squamous Non-Small-Cell Lung Cancer. N. Engl. J. Med..

[7] Socinski M.A., Jotte R.M., Cappuzzo F., Orlandi F., Stroyakovskiy D., Nogami N., Rodríguez-Abreu D., Moro-Sibilot D., Thomas C.A., Barlesi F. (2018). Atezolizumab for First-Line Treatment of Metastatic Nonsquamous NSCLC. N. Engl. J. Med..

[8] Hendriks L., Kerr K., Menis J., Mok T., Nestle U., Passaro A., Peters S., Planchard D., Smit E., Solomon B. (2023). Non-oncogene-addicted metastatic non-small-cell lung cancer: ESMO Clinical Practice Guideline for diagnosis, treatment and follow-up. Ann. Oncol..

[9] Jassem J., de Marinis F., Giaccone G., Vergnenegre A., Barrios C.H., Morise M., Felip E., Oprean C., Kim Y.-C., Andric Z. (2021). Updated Overall Survival Analysis From IMpower110: Atezolizumab Versus Platinum-Based Chemotherapy in Treatment-Naive Programmed Death-Ligand 1-Selected NSCLC. J. Thorac. Oncol..

[10] Sezer A., Kilickap S., Gümüş M., Bondarenko I., Özgüroğlu M., Gogishvili M., Turk H.M., Cicin I., Bentsion D., Gladkov O. (2021). Cemiplimab monotherapy for first-line treatment of advanced non-small-cell lung cancer with PD-L1 of at least 50%: A multicentre, open-label, global, phase 3, randomised, controlled trial. Lancet.

[11] Reck M., Rodríguez-Abreu D., Robinson A.G., Hui R., Csőszi T., Fülöp A., Gottfried M., Peled N., Tafreshi A., Cuffe S. (2016). Pembrolizumab versus Chemotherapy for PD-L1-Positive Non-Small-Cell Lung Cancer. N. Engl. J. Med..

[12] Nawaz K., Webster R.M. (2023). The non-small-cell lung cancer drug market. Nat. Rev. Drug Discov..

[13] Moliner L., Spurgeon L., Califano R. (2023). Controversies in NSCLC: Which second-line strategy after chemo-immunotherapy?. ESMO Open.

[14] Schiller J.H., Harrington D., Belani C.P., Langer C., Sandler A., Krook J., Zhu J., Johnson D.H. (2002). Comparison of Four Chemotherapy Regimens for Advanced Non–Small-Cell Lung Cancer. N. Engl. J. Med..

[15] Forde P.M., Spicer J., Lu S., Provencio M., Mitsudomi T., Awad M.M., Felip E., Broderick S.R., Brahmer J.R., Swanson S.J. (2022). Neoadjuvant Nivolumab plus Chemotherapy in Resectable Lung Cancer. N. Engl. J. Med..

[16] Planchard D. (2020). Adjuvant Osimertinib in *EGFR* -Mutated Non–Small-Cell Lung Cancer. N. Engl. J. Med..

[17] Felip E., Altorki N., Zhou C., Csőszi T., Vynnychenko I., Goloborodko O., Luft A., Akopov A., Martinez-Marti A., Kenmotsu H. (2021). Adjuvant atezolizumab after adjuvant chemotherapy in resected stage IB–IIIA non-small-cell lung cancer (IMpower010): A randomised, multicentre, open-label, phase 3 trial. Lancet.

[18] Shepherd F.A., Dancey J., Ramlau R., Mattson K., Gralla R., O’rourke M., Levitan N., Gressot L., Vincent M., Burkes R. (2000). Prospective Randomized Trial of Docetaxel Versus Best Supportive Care in Patients with Non-Small-Cell Lung Cancer Previously Treated with Platinum-Based Chemotherapy. J. Clin. Oncol..

[19] Fossella F.V., DeVore R., Kerr R.N., Crawford J., Natale R.R., Dunphy F., Kalman L., Miller V., Lee J.S., Moore M. (2000). Randomized Phase III Trial of Docetaxel Versus Vinorelbine or Ifosfamide in Patients with Advanced Non-Small-Cell Lung Cancer Previously Treated with Platinum-Containing Chemotherapy Regimens. J. Clin. Oncol..

[20] Reck M., Kaiser R., Mellemgaard A., Douillard J.-Y., Orlov S., Krzakowski M., von Pawel J., Gottfried M., Bondarenko I., Liao M. (2014). Docetaxel plus nintedanib versus docetaxel plus placebo in patients with previously treated non-small-cell lung cancer (LUME-Lung 1): A phase 3, double-blind, randomised controlled trial. Lancet Oncol..

[21] Hanna N., Shepherd F.A., Fossella F.V., Pereira J.R., De Marinis F., von Pawel J., Gatzemeier U., Tsao T.C.Y., Pless M., Muller T. (2004). Randomized Phase III Trial of Pemetrexed Versus Docetaxel in Patients with Non-Small-Cell Lung Cancer Previously Treated with Chemotherapy. J. Clin. Oncol..

[22] Herbst R.S., Khuri F.R., Lu C., Liu D.D., Fossella F.V., Glisson B.S., Pisters K.M.W., Shin D.M., Papadimitrakopoulou V.A., Kurie J.M. (2002). The novel and effective nonplatinum, nontaxane combination of gemcitabine and vinorelbine in advanced nonsmall cell lung carcinoma: Potential for decreased toxicity and combination with biological therapy. Cancer.

[23] Waechter F., Passweg J., Tamm M., Brutsche M., Herrmann R., Pless M. (2005). Significant Progress in Palliative Treatment of Non-small Cell Lung Cancer in the Past Decade. Chest.

[24] Lilenbaum R., Villaflor V.M., Langer C., O’Byrne K., O’Brien M., Ross H.J., Socinski M., Oldham F.B., Sandilac L., Singer J.W. (2009). Single-Agent Versus Combination Chemotherapy in Patients with Advanced Non-small Cell Lung Cancer and a Performance Status of 2: Prognostic Factors and Treatment Selection Based on Two Large Randomized Clinical Trials. J. Thorac. Oncol..

[25] Park S.E., Lee S.H., Ahn J.S., Ahn M.-J., Park K., Sun J.-M. (2018). Increased Response Rates to Salvage Chemotherapy Administered after PD-1/PD-L1 Inhibitors in Patients with Non-Small Cell Lung Cancer. J. Thorac. Oncol..

[26] Schvartsman G., Peng S.A., Bis G., Lee J.J., Benveniste M.F., Zhang J., Roarty E.B., Lacerda L., Swisher S., Heymach J.V. (2017). Response rates to single-agent chemotherapy after exposure to immune checkpoint inhibitors in advanced non-small cell lung cancer. Lung Cancer.

[27] Liu S., Hu X., Chirovsky D., Meng W., Samkari A. (2022). Overall Survival in Patients with Advanced NSCLC Receiving Taxane-Containing Regimen After Exposure to Immunotherapy and Platinum-Doublet. J. Thorac. Oncol..

[28] Auclin E., Benitez-Montanez J., Gorria T., Garcia-Campelo R., Dempsey N., Pinato D., Reyes R., Albarran V., Dall’Ollio F., Soldato D. (2022). OA07.06 Second Line Treatment Outcomes after Progression on Immunotherapy Plus Chemotherapy (IO-CT) in Advanced Non-small Cell Lung Cancer (aNSCLC). J. Thorac. Oncol..

